# Cost of a 5-year lung cancer survivor: symptomatic tumour identification *vs* proactive computed tomography screening

**DOI:** 10.1038/sj.bjc.6605253

**Published:** 2009-08-18

**Authors:** A W Castleberry, D Smith, C Anderson, A J Rotter, F W Grannis

**Affiliations:** 1 University of Pittsburgh School of Medicine, Pittsburgh, PA, USA; 2 Department of Thoracic Surgery, City of Hope National Medical Center, Duarte, CA, USA; 3 University of California San Diego, San Diego, CA, USA

**Keywords:** lung cancer, screening, comparative effectiveness research, computerised tomography, cost-effectiveness, QALY

## Abstract

**Background:**

Our objective was to analyse the cost effectiveness of computed tomography (CT) screening for lung cancer in terms of the cost per long-term survivor, which has not been evaluated to date.

**Methods:**

Estimations were computed based on data from the Surveillance, Epidemiology, and End Results registries covering years 1999–2003. The design framework of our model allowed for the incorporation of multiple values taken from the epidemiological and clinical literature to be utilised for cost inputs, scope of patients screened, diagnostic staging, and survival percentages applied separately to two cohorts: age 40–79 and 60–79 years. This enabled the analysis of over 1400 scenarios, each containing a unique set of input values, for which the estimated cost per 5-year survivor (CP5YS) was compared between the symptom-detected and proactive screening approaches.

**Results:**

Estimated CP5YS were higher for the symptom-detected approach in all 729 scenarios analysed for the cohort ages 60–79 years, ranging from approximately $5800 to $116 700 increased cost per 5-year survivor (CP5YS). For the cohort ages 40–79 years, 75% of the 729 scenarios analysed showed increased CP5YS for the symptom-detected approach ranging from $5700 to $110 000 increased CP5YS. Total costs and total 5-year survivors were higher for the proactive screening method for all scenarios analysed across both cohorts with increases ranging from 50–256% and 98–309%, respectively.

**Conclusion:**

The predicted increase in long-term survival with CT screening and the potential for better utilisation of health-care dollars in terms of CP5YS, particularly when screening patients over the age of 60 years, are critically important considerations in directing effective future lung cancer management strategy.

Lung cancer is the leading cause of cancer deaths nationwide. An estimated 162 000 people would have died of lung cancer in the United States in 2006 (American Cancer Society, 2006). Currently, only 16% of lung cancers are diagnosed when the disease is still localised and the 5-year survival for all stages combined is 15% (Jemal *et al*, 2005).

Because lung cancer is seldom symptomatic in early stage and treatment in advanced stage has very low survival (Mountain, 1997), current research has focused on methods to detect lung cancer at an earlier stage where curative treatment can be offered.

Results from the International Early Lung Cancer Action Project (I-ELCAP) demonstrating striking improvement in early stage detection of lung cancer by low-radiation-dose computed tomography (CT) screening (Henschke *et al*, 1999; Henschke *et al*, 2001) have sparked debate on the cost-effectiveness of a CT screening programme, with widely variant results reported (Marshall *et al*, 2001a; Marshall *et al*, 2001b; Chirikos *et al*, 2002; Mahadevia *et al*, 2003; Wisnivesky *et al*, 2003). In a systemic review published in January 2006 (Black *et al*, 2006), the authors conclude that many issues remain unresolved in the debate over the true cost-effectiveness of lung cancer screening programmes. Attention to this issue has further escalated following the reported results of over 30 000 persons screened in the large collaborative I-ELCAP study, which concluded that annual spiral CT screening can detect lung cancer that is curable in most cases (Henschke *et al*, 2006a). In addition, an analysis of the mean sojourn time and sensitivity of CT screening for lung cancer estimated approximately 23% mortality reduction possible by an annual CT screening programme opposed to observation (Chien and Chen, 2008). Knudsen *et al* (2007) have emphasised the need for mathematical models that examine the cost effectiveness of implementation of population CT screening.

Many investigations of cancer treatment cost-effectiveness utilise quality-adjusted life year (QALY) as the primary metric to measure the results of interventions that provide some improvement in quality and/or duration of life, but only for limited periods of time. For example, a cancer treatment that results in improvement in duration of survival by 3 months at cost of $25 000 would result in a cost per year or QALY of approximately $100 000, as it requires four patients, each experiencing a 3-month improvement in survival to add up to a year of life gained. Although this metric has the advantage of wide utilisation, in a certain sense, it is a misleading figure, as in this example no single patient would actually survive 1 year. Preventive screening measures, where the positive impact on health outcomes may not be measurable for many years, may be difficult to quantify using QALYs (Phillips and Thompson, 2003). Arguably, the most important consideration in determining cost effectiveness for differing approaches to lung cancer is the number of long-term survivors attained by a given strategy. Accordingly, we believe that the cost of a long-term survival is more meaningful to the reader, and is particularly more important to patients at high-risk of lung cancer. The purpose of this study is to evaluate the estimated cost-effectiveness of a proactive screening programme compared with current lung cancer strategies when applied to a representative subset of the US population. We compared the ratio of 5-year survivors to the total screening, diagnosis, and treatment costs for the current management algorithm that we will designate as the symptomatic tumour identification (STID) approach and for an early detection approach using CT screening (EDCTS). Second, we report the estimated total costs and total 5-year survivors associated with each strategy. The EDCTS protocol is based on I-ELCAP recommendations (Henschke, 2006). We separately applied this analysis to two cohorts distinguished by age. One cohort was in the age of 40–79 years and the second cohort was in the age of 60–79 years.

## Materials and methods

We constructed a mathematical model based on multivariate analysis of empirical data to estimate the ratio of screening, diagnosis, and treatment costs per 5-year non-small-cell lung cancer (NSCLC) survivor for two different lung cancer management strategies, STID and EDCTS. These estimations were computed in 2005 USD based on the most recent 5 years of data available, extending from 1999 to 2003, within the 13 Surveillance, Epidemiology, and End Results (SEER) data registries (National Cancer Institute, 2005). With respect to selected demographic and epidemiologic factors, these areas are a reasonably representative subset of the US population (Ries *et al*, 2005). The scope of our analysis covered a population of approximately 15 million people.

The design framework of our model allowed for the incorporation of multiple values taken from the epidemiological and clinical literature to be utilised for cost inputs, scope of patients screened, diagnostic staging, and survival percentages. This methodology enabled the analysis of over 700 scenarios for each of the two cohorts (for a total of over 1400 scenarios), each containing a unique set of input values. For each of these scenarios, the estimated CP5YS was compared between the STID and EDCTS approaches. By analysing a broad range of parameter values, we were able to evaluate the sensitivity of selected variables by using a range of favourable and unfavourable estimates. In addition, this study design also enabled the evaluation of completely separate parameter estimates and calculations from different sources of published data. Our model assumes that the capital equipment and resources necessary for both management strategies are already in place.

### Input variables and source data

*Population screened*. The initial I-ELCAP screening study published in 1999 screened at-risk persons aged 60 years or older (Henschke *et al*, 1999). The subsequent large collaborative I-ELCAP study published in 2006 screened at-risk persons aged 40 and older (Henschke *et al*, 2006a). Approximately 98.3% of the 31 567 persons screened in the large collaborative I-ELCAP study were under the age of 80 years. As such, we analysed two separate cohorts: one cohort was of the age 40–79 years and the second cohort was of the age 60–79 years. The population screened for lung cancer in our analysis was based on inclusion criteria from I-ELCAP reported studies, which screened patients with a history of at least 10 pack-years of cigarette smoking, no history of cancer (other than non-melanoma skin cancer), and who were fit to undergo thoracic surgery (Henschke *et al*, 1999). We estimated this population using the following steps:

First, we determined the total population per the 13 SEER data registries from 1999–2003 for each of the two cohorts. This total population was reduced by 0.45% to reflect the incidence of cancer other than non-melanoma skin cancer per the SEER Cancer Statistics Review (Ries *et al*, 2005).

Second, we estimated the percentage of US adults in each cohort with at least a 10 pack-year history of smoking, regardless of current smoking status, using figures reported by the 2003 National Cancer Institute and Centers for Disease Control and Prevention Co-sponsored Tobacco Use Special Cessation Supplement to the Current Population Survey (CPS) (National Cancer Institute, 2003). To evaluate the sensitivity of this parameter and incorporate a range of variability in actual smoking history, a 10% increase/decrease in the CPS percentage was also analysed as upper and lower bound estimates, respectively (Table 1).

We then estimated the percent of this population that would be unfit for surgery by using SEER data to determine the percent of stage I tumours diagnosed in patients aged 40–79 and 60–79, respectively for each cohort, in whom surgery was not recommended or was contraindicated (Criteria: lung and bronchus NSCLC, invasive, microscopically confirmed, the patient's first primary cancer, and diagnosed between 1999 and 2003. Cases where surgery was recommended and the patient refused the procedure were counted the same as cases where surgery was performed. Cases where it was unknown whether surgery was recommended were omitted from the analysis). This resulted in an estimate of 14.7% for the cohort ages 40–79 years who are excluded from screening because they would not be fit for surgery and 17.0% for the cohort ages 60–79 years. It is important to emphasise that some individuals who would not be the candidates for standard surgical procedures might still receive potentially curative treatment with minimally invasive surgical approaches, radiation therapy, radiofrequency ablation, or other methods.

Using steps (1), (2), and (3) described above, we quantified an estimate of the total population that would be eligible for screening in each cohort in accordance with I-ELCAP inclusion criteria, with an upper and lower bound estimate for the percent of the population with a 10 pack-year history of smoking (Table 1). Statistics on compliance with breast cancer screening guidelines from three separate studies (Horton *et al*, 1996; Phillips *et al*, 1998; Rahman *et al*, 2003) were used to estimate the compliance with lung cancer screening recommendations (Table 1). Multiplying the SEER population meeting the inclusion criteria by the estimated compliance percent provides estimates of the total population that would receive the screening procedures (Table 2).

The choice of different inclusion criteria (e.g., older age at entry, or higher/lower pack-years requirement) would result in changes in number of individuals screened, costs, and results.

#### Baseline screening vs annual repeat screening.

In the I-ELCAP studies, the percentage of patients requiring additional tests subsequent to the initial CT scan was different in the first year (baseline screen) than in annual repeat screening. In addition, the percent of cases where malignancy was found was also different for baseline screens *vs* annual repeat screens (Henschke *et al*, 1999; Henschke *et al*, 2001; Henschke *et al*, 2006a). For the purpose of our estimation of screening costs and malignancies detected, the ‘Population Screened’ described above for each year was divided into groups reflecting those receiving a baseline screen *vs* those receiving repeat annual screens. This was carried out based on the large collaborative I-ELCAP study, in which 31 567 persons received a baseline screen with 27 456 (87.0%) following up with an annual repeat screen (Henschke *et al*, 2006a). This reflects a dropout rate (i.e., the percent of patients who receive a baseline scan and do not follow up with annual repeat scans) of 13%. This information was used to estimate baseline and repeat screens as follows: (1) in year one, all screens are baseline screens; (2) in all subsequent years, 87.0% of the patients screened in the earlier year are assumed to receive repeat screens with the remaining patients screened in that year assumed to be new patients receiving a baseline screen (see Table 3).

#### Frequency and type of screening procedures carried out.

The procedures carried out as part of the baseline and annual repeat screens were based on I-ELCAP protocol, revision dated 20 October 2006 (Henschke, 2006). The I-ELCAP recommendations for the diagnostic workup in participants with a positive result on the CT include multiple options for nodules meeting certain size criteria. The protocol used in the I-ELCAP research was reviewed semi-annually and modified over time and with increasing experience in the analysis of the study data, which likely impacted the volume of procedures indicated in years subsequent to the baseline year because of a temporal, learning effect. In addition, in the I-ELCAP studies, the decision regarding how to proceed is left to each participant and the referring physician (Henschke *et al*, 2006a). As such, the screening procedure protocol utilised in this analysis (referred to as ‘EDCTS Screening Protocol’) may not precisely reflect the I-ELCAP protocol or screening procedures presented in I-ELCAP studies. The EDCTS Screening Protocol identifies additional workup procedures which, based on the size of nodules identified in the initial CT scan, would be included in a patient's screening costs. We derived the percentage of total screened patients receiving each additional workup procedure based on the size distribution of screen tumours reported in the I-ELCAP baseline and repeat screening studies (Henschke *et al*, 1999; Henschke *et al*, 2001) (Tables 4 and 5). A summary of the percent of screening participants receiving each type of screening procedure is provided in Table 1. Workup procedures that also involve treatment of the malignancy, such as video-assisted thoracoscopic surgical biopsy, are considered as treatment procedures and are not included as part of screening procedures in this analysis.

#### Screen-detected malignant tumours.

For the cohort ages 60–79 years, the number of malignant tumours identified by screening in the baseline and annual repeat screens was derived by multiplying the percent of malignant tumours identified in the I-ELCAP studies published in 1999 and 2001 that screened patients aged 60 years and over (Henschke *et al*, 1999; Henschke *et al*, 2001) by the total population screened (Table 1). For the cohort ages 40–79 years, the percentage of malignant tumours identified in the large collaborative I-ELCAP study published in 2006 that screened patients aged 40 years and over was used (Henschke *et al*, 2006a) (Table 1).

#### Screening costs.

For the EDCTS approach, our model incorporated three different screening procedure cost estimates for multidetector CT (MDCT) scans and fine-needle aspiration biopsies (FNAB), and two cost estimates for positron emission tomography (PET) scans taken from the clinical literature (published costs for conventional CT scans were used for MDCT costs, as there is typically no difference in the cost to the payor). The first set of estimates for CT and FNAB were based on the actual costs incurred by I-ELCAP volunteers, as recorded by the New York-Presbyterian Hospital's financial system cost database (Wisnivesky *et al*, 2003). The second and third sets of estimates were based on 2003 published figures from a cost effectiveness study by Mahadevia *et al* (2003). The Mahadevia *et al* (2003) study reported base-case costs for lung cancer screening procedures as well as favourable and unfavourable extremes. Values from both the favourable and unfavourable scenarios were utilised in our model (Table 1). The first estimate of PET scan costs was taken from 2005 Medicare Reimbursement rates, and the second cost estimate (unfavourable screening estimate) was taken from a 2004 published cost-effectiveness study by Kelly *et al* (2004) (Table 1). We did not consider the potential costs or implications of using PET-CT, as this was not included in the EDCTS Screening Protocol (see above). All costs were calibrated to 2005 US dollars using the medical component of the consumer price index. Screening costs for the STID method were assumed to be zero.

#### Quantity and distribution of tumours by stage.

Tumour quantity and distribution by stage for the STID method was based on SEER data from 1999 to 2003, using lung and bronchus NSCLC that were invasive, microscopically confirmed, and the patient's first primary cancer. SEER tumours of unknown stage were allocated to stages based on the distribution percentages of the staged SEER tumours.

The quantity of tumours for the EDCTS method was comprised of two components: (1) screen-detected malignant tumours (quantification described above); and (2) non-screen-detected tumours (‘symptomatic tumours’). For the first year of screening implementation it was assumed that the number of symptomatic tumours would include all SEER reported tumours for that year. For subsequent years, symptomatic tumours were calculated by multiplying the percent of the population that did not comply with screening recommendations (based on estimates described for ‘Population Screened’ above) to the SEER tumour population. For example, if the SEER data identified 1000 tumours in a given year, and an estimated 60% of the population did not comply with screening recommendations, then it was assumed that 600 (=1000 × 60%) symptomatic tumours would be recorded in that year in addition to screen-detected tumours. Components (1) and (2) were added together to determine the total EDCTS tumours identified each year.

The stage of EDCTS tumours was determined as follows: (1) Symptomatic tumours, as described above, maintain the SEER stage distribution, with the same treatment for tumours of unknown stage as described above for the STID method. (2) Three estimations were used to determine the number of remaining screen-detected malignant tumours allocated to each stage. The first estimation was based on I-ELCAP data (Henschke *et al*, 1999), the second was based on a prospective cohort CT screening study by Swensen *et al* (2002), and the final estimation was an average of the I-ELCAP and Swensen stage distribution percentages (Table 1).

#### Cost of treatment by stage.

For this parameter, we included separate estimates and calculations from different sources of published data on the cost of diagnosis and treatment for lung cancer by stage. These sources included data based on Medicare payments for lung cancer patients in nine SEER registries (Riley *et al*, 1995), cost of medical care for patients of a Northern California health maintenance organisation (Fireman *et al*, 1997), and costs under Canada's universal health-care system (Evans *et al*, 1995). A previous study by Wisnivesky *et al* (2003) noted that the overall distribution of the costs of lung cancer treatments by stage were similar in the United States and Canada. These estimates included the total lung cancer cost by stage from diagnosis to death, adjusted to 2005 US dollars (Table 1).

#### Total screening, diagnosis, and treatment costs.

For the EDCTS approach, total costs were comprised of screening cost and the cost of treatment by stage. However, calculations for the cost of treatment by stage included diagnosis and staging procedures that are also covered in the screening protocol. To avoid double-counting these expenditures, the screening costs for patients diagnosed with malignant lung cancer were removed from the total costs, as these costs are already included in the cost of treatment by stage. As such, the resulting total costs for the EDCTS approach includes the sum total of screening costs for patients not diagnosed with malignant cancer (over 97% of total screening costs) and the cost of treatment by stage (including diagnostic costs) for the entire tumour population, including screen-detected and non-screen-detected tumours. Total costs for the STID approach were limited to the cost of treatment by stage with screening costs assumed to be zero.

#### Five-year survivors.

Survival rates were applied to the population of tumours by stage to determine the estimated number of associated 5-year survivors. The same survival rates were used for both the STID and EDCTS calculations. Three different sets of survival rate estimates were included in our analysis. The first set of estimates was based on published data on over 5300 lung cancer cases compiled from The University of Texas MD Anderson Cancer Center and the classification research database from the Reference Center for Anatomic and Pathologic Classification of Lung Cancer (survival based on death from cancer or unknown cause) (Mountain, 1997). For the second set of estimates, stage I survival was based on I-ELCAP 10-year survival results (lung cancer-specific survival; 10-year survival data were used as a conservative estimate of 5-year survival) (Henschke *et al*, 2006a), with stage II through stage IV based on 713 043 primary lung malignancies submitted to the National Cancer Data Base (relative survival) (Fry *et al*, 1999). The final set of estimates used the same National Cancer DataBase rates for stage II through stage IV disease, with the stage IA survival rate from a 2000 study published by Patz *et al* (2000), applied to the stage I tumour populations (all-cause survival) (Table 1).

### Ratio of total CP5YS for 729 scenarios

**Figure 1:**
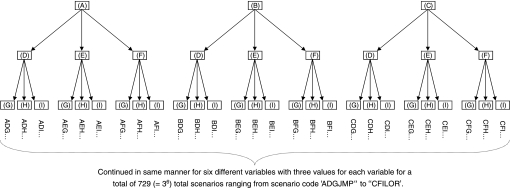
Illustration of the method of using multiple input value combinations used to generate 729 scenarios for each cohort. Identification codes (A), (B), and (C) represent the three input values for the percent of US adult smokers from Table 1. Similarly, (D), (E), and (F) represent three input values for percent complying with screening recommendations, and (G), (H), and (I) represent the three input values for screening procedure costs. ‘Scenario code’ refers to the combination of input value identifiers for multiple variables.

Using the inputs described above, 729 scenarios were generated by combining all possible combinations of the variables containing multiple input values in Table 1. For example, the percent of the population eligible for screening (⩾10 pack-year history of smoking) has three possible input values: (A), (B), and (C). Similarly, the estimated compliance percentage has three possible input values: (D), (E), and (F). Each combination of these values was used in a separate scenario examined (AD, AE, AF, BD, and so on). This method was applied to all six variables that contain multiple input values (three input values each) for a total of 729 (=3^6^) total scenarios for each cohort analysed (Figure 1). Each unique combination of input values was given a six letter ‘scenario code’ based on the combination of input identifiers utilised. For example, the scenario code of ADGJMP represents a scenario in which estimate (A) was used for the percent of US adults with a 10 pack-year history of smoking (28.4%), estimate (D) was used for the compliance with screening recommendations (27%), estimates (G) were used for screening procedure costs, and so on.

**Figure 2:**
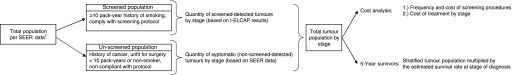
Schematic illustration of the calculation of costs and 5-year survivors for the early detection computed tomography screening (EDCTS) method. Costs and 5-year survivors for the symptomatic tumour identification (STID) method follow the path of the ‘un-screened population’ above. ^†^The calculation exemplified by the above schematic was separately carried out for age range 40–79 years and age range 60–79 years.

The total costs, total 5-year survivors, and the ratio of total cost to 5-year survivors were analysed for all 1458 scenarios (729 scenarios for each cohort). The CP5YS ratio was determined by first calculating the estimated number of NSCLC tumours distributed by stage for the 5-year period for both the STID and EDCTS approaches. The cost of diagnosis and treatment by stage were applied to these tumour populations, with screening cost also added to the EDCTS costs. The estimated percent of 5-year survivors for each stage was also applied to the tumour populations. The ratio of total CP5YS was then calculated (see Figure 2).

## Results

Note that the total survivors and total cost figures reported in this analysis refer only to the subset of the US population studied and are not extrapolated to represent nationwide estimates.

### Cohort ages 40–79 years

**Figure 3:**
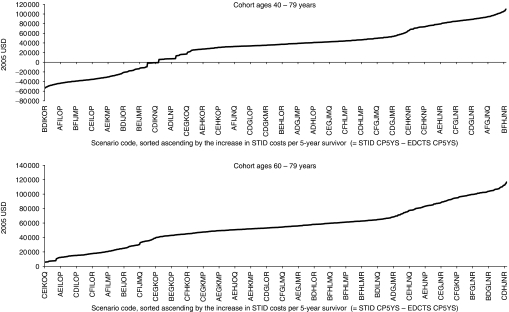
Increase in STID costs per 5-year survivor (CP5YS) compared with the EDCTS method (STID CP5YS–EDCTS CP5YS). The scenario codes represent the combination of input variables and associated codes from Table 1. All 729 scenario codes are represented in each graph, though not all *Y* axis values are labelled. Note that the data are not sorted alphabetically by the *X* axis scenario codes. STID=symptomatic tumour identification; EDCTS=early detection approach using computed tomography screening. Note: Across all scenarios, the total number of 5-year survivors was higher for the EDCTS method, ranging from approximately 13 400 to 39 300 increased 5-year survivors for the population aged 40–79 years and 9300–27 400 for the population aged 60–79 years.

**Figure 4:**
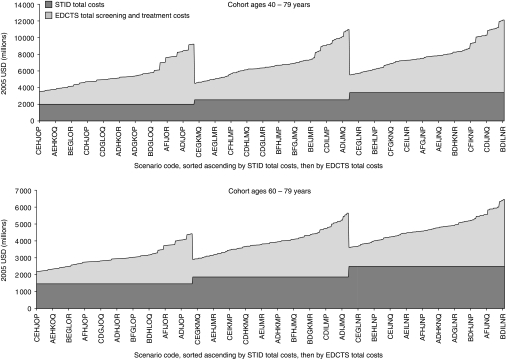
Comparison of the total screening, diagnosis, and treatment costs between the STID and EDCTS methods. The scenario codes represent the combination of input variables and associated codes from Table 1. All 729 scenario codes are represented in each graph, though not all *Y* axis values are labelled. Note that the data are not sorted alphabetically by the *X* axis scenario codes. STID=symptomatic tumour identification, EDCTS=early detection approach using computed tomography screening.

Across all 729 scenarios analysed for the cohort ages 40–79 years, the total CP5YS ranged from $157 300 to $293 800 for the STID method, and from $105 100 to $288 100 for the EDCTS method. The costs per 5-year survivor were higher for the STID approach in 75% of the scenarios analysed, ranging from approximately $5700 to $110 000 increased CP5YS under the STID method for those scenarios (Figure 3) (See the Appendix Table A1 for a listing of the CP5YS for all 729 scenarios). The total number of 5-year survivors over the 5-year period analysed was higher for the EDCTS method for all 729 scenarios, ranging from approximately 13 400 to 39 300 increased 5-year survivors under the EDCTS method, which is a 112–309% increase. The total screening, diagnosis, and treatment costs were also higher for the EDCTS method in all 729 scenarios, ranging from approximately $1.5B to $8.7B in increased total costs, which is a 77–256% increase (Figure 4).

The EDCTS method resulted in a 27–66% increase in the number of tumours identified when compared with the STID method (Table 6).

Scenario code BDHJOQ was associated with the lowest CP5YS of approximately $105 100 per 5-year survivor for the EDCTS method and $157 300 for the STID method. Scenario code CEIKNP was associated with the highest CP5YS of approximately $288 100 per 5-year survivor for the EDCTS method and $293 800 for the STID method.

The scenario most favourable towards the EDCTS method in terms of the comparative decrease in costs per 5-year survivor was scenario code BDHJNR, in which the EDCTS CP5YS was approximately $110 000 less than the STID ratio for that same scenario (305% more EDCTS 5-year survivors in that scenario). The scenario least favourable towards the EDCTS method in terms of the comparative increase in costs per 5-year survivor was scenario code BDIKOR, in which the EDCTS CP5YS was approximately $53 400 more than the STID ratio for that same scenario (248% more EDCTS 5-year survivors in that scenario).

### Cohort ages 60–79 years

Across all 729 scenarios analysed for the cohort ages 60–79 years, the total CP5YS ranged from $149 400 to $282 100 for the STID method, and from $86 400 to $233 300 for the EDCTS method. The costs per 5-year survivor were higher for the STID approach in all scenarios analysed, ranging from approximately $5800 to $116 700 increased CP5YS under the STID method for those scenarios (Figure 3) (See the Appendix Table A2 for a listing of the CP5YS for all 729 scenarios). The total number of 5-year survivors over the 5-year period analysed was higher for the EDCTS method for all 729 scenarios, ranging from approximately 9000 to 26 600 increased 5-year survivors under the EDCTS method, which is a 98–272% increase. The total screening, diagnosis, and treatment costs were also higher for the EDCTS method in all 729 scenarios, ranging from approximately $728 M to $4.0B in increased total costs, which is a 50–159% increase (Figure 4).

The EDCTS method resulted in a 24–60% increase in the number of tumours identified when compared with the STID method (Table 6).

Scenario code BDHJOQ was associated with the lowest CP5YS of approximately $86 400 per 5-year survivor for the EDCTS method and $149 400 for the STID method. Scenario code CEIJNP was associated with the highest CP5YS of approximately $233 300 per 5-year survivor for the EDCTS method and $282 000 for the STID method.

The scenario most favourable towards the EDCTS method in terms of the comparative decrease in costs per 5-year survivor was scenario code BDHJNR, in which the EDCTS CP5YS was approximately $116 700 less than the STID ratio for that same scenario (269% more EDCTS 5-year survivors in that scenario). The scenario least favourable towards the EDCTS method in terms of the comparative decrease in costs per 5-year survivor was scenario code CEIKOQ, in which the EDCTS CP5YS was approximately $5800 less than the STID ratio for that same scenario (98% more EDCTS 5-year survivors in that scenario).

## Discussion

The advisability of population screening for lung cancer is currently a topic of active debate. Because of the potential high cost of lung cancer screening programmes, cost-effectiveness questions are an important component to be evaluated. This article attempts to inform the debate by a multivariable analysis model. We incorporated a range of optimistic and pessimistic input variables taken from literature sources on both sides of this debate. In addition, this analysis presents an important method for evaluating a lung cancer-screening programme in contrast to analyses using QALYs, which can be misleading in terms of the efficacy of treatment regimens, particularly when there is little chance of long-term survival, as in the treatment of stage IIIB and IV NSCLC with chemotherapy. Owing to the frequent use of the QALY metric in the literature, we have provided for the reader a cost-per-year of life calculation for all 729 scenarios in the Appendix Table A3 and Table A4 to allow a rough comparison to other studies evaluating cost-effectiveness on a per-year basis. It should be emphasised, however, that the data in Table A3 and Table A4 is calculated assuming that these lung cancer survivors live only 5 years. It is therefore a conservative estimate and overstates the cost-per-year of life, as many lung cancer survivors detected by screening will live longer than 5 years (Marcus *et al*, 2000; Sobue *et al*, 2002; Henschke *et al*, 2006a).

The combination of decreased CP5YS and increased 5-year survivors in all 729 scenarios for the cohort ages 60–79 years and in the majority of scenarios for the cohort ages 40–79 years suggests that health-care dollars spent on proactively screening for lung cancer may achieve higher cost-effectiveness. These data may also suggest advantages in focusing a screening programme on patients aged 60 years or older as the marginal costs for implementing screening in the cohort ages 60–79 years were between 46 and 60% of the marginal cost for the cohort ages 40–79 years, with greater cost effectiveness in the 60–79 age group when compared with the STID method (see Figure 3). These findings are consistent with a 2008 study by Whynes (2008), analysing the potential cost effectiveness of lung cancer screening in the United Kingdom.

**Figure 5:**
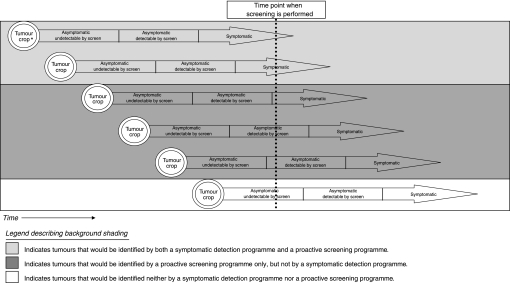
Illustration demonstrating the theory for an increase in the number of tumours identified by screeingfor asymptomatic malignancies in addition to identifying symptomatic tumours. ‘Tumour crop’ refers to a cohort of lung cancers that clinically manifested at a point in time (i.e., a group of tumours for which the tumours' inception is at the same time point represented on the *X* axis).

The I-ELCAP study results have shown the ability of CT scanning to detect asymptomatic lung cancers. By definition, a lung cancer programme that detects symptomatic and asymptomatic malignancies will diagnose more tumours at the onset of the programme than a system that detects symptomatic tumours alone, as illustrated in Figure 5. The magnitude of this increase is unknown, and sufficient information to evaluate the reasonableness of the 24–66% increase in diagnosed tumours under the EDCTS method presented in this model is currently unavailable. Additional long-term data on the prevalence of lung cancer in an asymptomatic at-risk population annually screened over multiple years are needed to enable further estimates in this area.

Increased total costs associated with a successful proactive screening programme are to be expected, not only due to the cost of screening procedures, but also more importantly due to the increased patient survival and related increase in long-term treatment costs and follow-up care. The available data for cost of treatment utilised in this study (see Table 1, inputs M, N, and O) all indicate that the treatment for early stage cancer is more expensive than cancers detected in stages IIIB and IV. This is counter-intuitive, as stage I NSCLC is treated by surgery alone, whereas advanced stage lung cancers require multimodality treatment, adding the cost of expensive radiation and chemotherapy. Furthermore, treatment of advanced stage lung cancer is changing, which may potentially shift this balance, making the treatment of late-stage cancers increasingly more expensive. The percentage of patients in higher stages who are receiving expensive radiation therapy and chemotherapy appears to be increasing (Ramsey *et al*, 2004; Langer *et al*, 2005). In addition, new molecular ‘targeted’ medications used in second- and third-line treatment may markedly increase treatment costs (Adis International Ltd. Erlotinib, 2003). As I-ELCAP data report increased actuarial 10-year survival of 80% with screen-detected tumours (Henschke *et al*, 2006a), which is dramatically different than the survival of symptom-detected tumours which are disproportionately detected in later stages, it would follow that increased costs of long-term care would be expected. The cost of follow-up and care in the majority of survivors, however, adds little to routine health-care expenditures, and only a small minority would require downstream salvage or palliative radiation therapy and chemotherapy. In addition, the cost of screening programmes and treatment may reasonably be expected to decrease as a result of economies of scale, if lung cancer screening were implemented at a state or nationwide level.

It is important to consider several potential sources of bias when evaluating lung cancer screening:

(1) ‘*Overdiagnosis bias*’: small, slow-growing lesions are detected by screening for intervention that would never become symptomatic within a patient's lifetime in the absence of screening (Black *et al*, 2006). This could be caused by an improper pathological diagnosis; however, all lung cancers in the I-ELCAP study are vetted by a panel of prominent international pathologists (Flieder *et al*, 2006). With regard to the theoretical possibility of screening the detection of very slow growing malignant neoplasms that do not cause symptoms during the patient's anticipated normal lifespan, for ethical reasons, a randomised trial comparing surgery with no surgery for stage I NSCLC is not possible. However, data on untreated screen-detected NSCLC from screening studies including the Johns Hopkins study, the Memorial Sloan-Kettering study, the Mayo Clinic study, and the I-ELCAP study indicate that almost all of these untreated patients die within 5 years (Flehinger *et al*, 1992; Henschke *et al*, 2006a). A study by Raz *et al* (2007), examined the natural history of patients with stage I NSCLC and concluded that long-term survival with untreated stage I NSCLC is uncommon with an overall survival of 6% for untreated stage I NSCLC. In addition, a 2003 study by Henschke *et al* analysed data from 885 cases of stage IA lung cancer and concluded that almost all diagnosed cases of Stage IA lung cancer have a malignant natural course, fatal if not treated, thus representing genuine cancer. Although some speculate that the explanation for the paradoxical result in the Mayo Lung Trial of increased survival but no reduction in mortality with chest radiograph screening was due to, in part, overdiagnosis bias (Patz, 2006), no empirical evidence to support this theory exists. Furthermore, Yankelevitz *et al*, (2003) analysed the doubling time of stage I tumours in the Mayo Lung Trial and concluded that ‘the hypothesis that early-stage lung tumours diagnosed on chest radiography during lung carcinoma screening may frequently be overdiagnosed, indolent cases needs to be rejected’.

Another form of overdiagnosis could exist in instances where comorbid disease would kill the patient before symptoms of lung cancer were experienced. This form of overdiagnosis bias can be reasonably assumed to be rendered largely irrelevant in medical environments, where patients are managed with good, sensible clinical judgment by physicians adept in identifying comorbid disease and reasonably accurately estimating the anticipated survival of their patients. Furthermore, in this study we have carefully taken into account the percentage of patients eligible for screening who have serious comorbid disease and excluded them from our analysis (refer to the ‘Population Screened’ section of the Materials and Methods above). We have been conservative in this approach by additionally excluding patients aged 80 years and above from screening, although many 80-year-olds are perfectly capable of undergoing minimally invasive surgical resection of lung cancers or being treated with radiationtherapy.

Finally, overdiagnosis could result if spontaneous remission of a preclinical cancer were to occur. Case reports of this phenomenon are extremely rare (Kappauf *et al*, 1997).

(2) ‘*Length bias*’: detection of more patients with less aggressive disease and fewer of those with more aggressive disease, because the duration of asymptomatic disease is longer in less aggressive tumours (Black *et al*, 2006). This also could result in ‘overdiagnosis’, discussed above. The baseline round of screening is inherently different from the repeat rounds because cancers with a longer latent (asymptomatic) phase are more frequently identified in the baseline round, whereas cancers found in repeat rounds are found earlier in their latent phase than in the baseline round (Morrison, 1982; Henschke and Yankelevitz, 2008). Cancers that are diagnosed at baseline, thus, tend to grow more slowly than does the subtype in general; they also grow more slowly than do tumours that are diagnosed in repeated screenings. As noted by the I-ELCAP researchers, this fact does not introduce a bias, but it may call for making a distinction between baseline screening and repeated screening (Henschke *et al*, 2007). ‘Length bias’ also implies that the faster growing tumours may present symptomatically between screening exams, however, the I-ELCAP data showed a very low incidence of such cases.

(3) ‘*Lead-time bias*’: screening-detected patients are accorded extended survival times solely because cancer was detected earlier owing to screening, although death occurred at the same time as would have happened without screening (Black *et al*, 2006). To address this potential source of bias, the estimated percentage of stage I 5-year survivors for all scenarios using input variable ‘Q’ (see Table 1) are based on 10-year survival percentages reported by I-ELCAP (Henschke *et al*, 2006a). If lead-time bias was evident in this study, a large number of individuals who survived 5 years would be expected to die before 10 years; the I-ELCAP survival curve shows no decrement in survival between 5 and 10 years (Henschke *et al*, 2006a).

Although some emphasise that the risks and complications of lung cancer screening may be considerable (Bach *et al*, 2007), the minutes of National Cancer Institute's National Lung Screen Trial (NLST) Data Safety Monitoring Board (DSMB), which we have reviewed, indicate that during the first 5 years of the study, which began in 2002, no unanticipated complications or risks have been recognised. The DSMB, which is responsible for the safety of NLST research subjects, has neither terminated the study nor reported information on new complications to study subjects.

### Limitations

Input variables based on SEER data may not be representative of actual nationwide lung cancer incidence or staging. Our model does not account for costs associated with complications from biopsies or other screening procedures, nor does it account for the increase in capital equipment and resources necessary to implement large-scale comprehensive CT screening programmes. In addition, our study design does not consider the indirect cost of lost productivity attributable to lung cancer morbidity and mortality. Of the estimated $167 billion costs of all diseases caused by tobacco products, indirect costs ($92 billion) are substantially higher than direct costs (Centers for Disease Control and Prevention, 2005). As survival increases, indirect costs attributable to loss of patient income, spousal income, and other factors may reasonably be expected to diminish substantially. This study is based on a representative subset of the US population and it is beyond the scope of this analysis to extrapolate the results to a state or nationwide level. The model does not factor in any additional benefit conferred by survival beyond 5 years. On the basis of the results from the Mayo Lung Trial (Marcus *et al*, 2000), Japanese Anti-Lung Cancer Association (ALCA) trial (Sobue *et al*, 2002), and I-ELCAP (Henschke *et al*, 2006a), there is a strong evidence to suggest that the majority of 5-year survivors will continue to survive for 10, 15, and even 20 years following diagnosis and treatment. Finally, there is no accurate method to calculate a dollar value for not dying of lung cancer in an individual or group of individuals with lung cancer.

## Conclusion

Our analytical model offers an innovative tool that provides data estimates that contribute insights into the continuing debate on the wisdom and advisability of implementing state and/or nationwide CT screening programmes. The predicted increase in long-term survival with CT screening and the potential for better utilisation of health-care dollars in terms of CP5YS, particularly when screening patients over the age of 60 years, are critically important considerations in directing effective future lung cancer management strategy.

## Figures and Tables

**Table 1:**
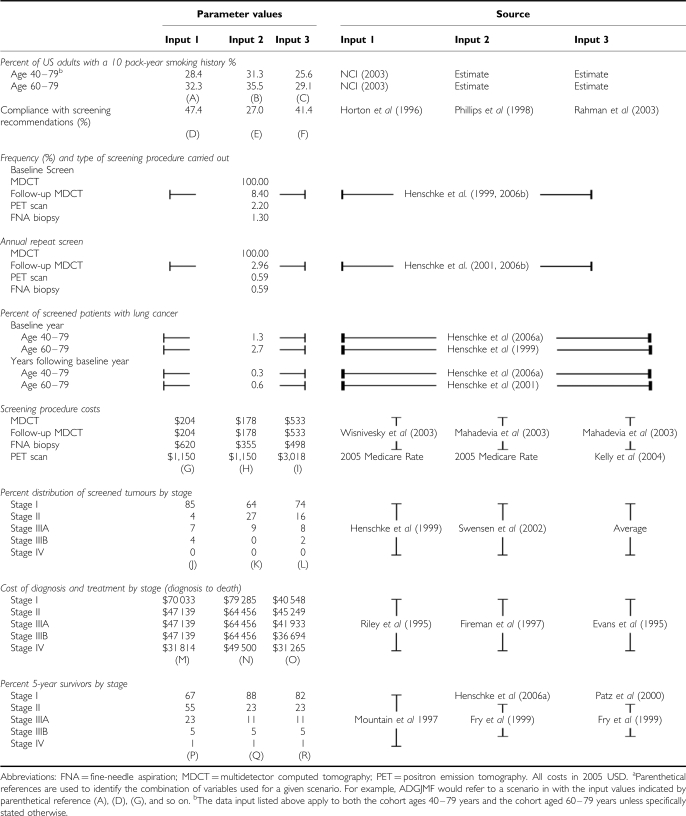
Summary of input variables and source data^a^

**Table 2:** SEER population estimated to receive screening for lung cancer based on inclusion criteria from I-ELCAP reported studies, nationwide smoking history statistics, and estimated compliance with screening recommendations^a^

	**Year 1**	**Year 2**	**Year 3**	**Year 4**	**Year 5**
SEER population age 40–79 years^b^	14 277 295	14 596 718	14 944 483	15 262 435	15 578 827
Less: cancer other than non-melanoma skin cancer (see text)	64 248	65 685	67 250	68 681	70 105
	14 213 047	14 531 033	14 877 233	15 193 754	15 508 722
Less: estimated patients unfit for surgery (see text)	2 089 318	2 136 062	2 186 953	2 233 482	2 279 782
	12 123 729	12 394 971	12 690 280	12 960 272	13 228 940
Less: <10 pack-year smoking history or nonsmoker (see text)^a^	8 675 948	8 870 053	9 081 381	9 274 592	9 466 855
	3 447 782	3 524 918	3 608 899	3 685 680	3 762 085
Less: non-compliance with screening recommendation (see text)^a^	1 813 533	1 854 107	1 898 281	1 938 668	1 978 857
Total screened population	1 634 248	1 670 811	1 710 618	1 747 012	1 783 228

a The values shown above for 10 pack-year smoking history and compliance with screening recommendations are based on the ‘Input 1’ column in Table 1. For simplicity, values generated using the columns labelled ‘Input 2’ and ‘Input 3’ values are not shown here.

b For simplicity, the values associated with the cohort ages 60–79 years are not shown here.

**Table 3:** Breakdown of patients receiving a baseline screen *vs* repeat annual screen in each year analysed

	**Year 1**	**Year 2**	**Year 3**	**Year 4**	**Year 5**
(A) Total screened population Age 40–79 (see Table 2)^a^,b	1 634 248	1 670 811	1 710 618	1 747 012	1 783 228
					
*Breakdown of the above total population screened (A) into baseline screens and annual repeat screens based on assumptions outlined in the section ‘Baseline screening vs annual repeat screening’ (see text):*					
(B) Dropouts to screening in subsequent years (=13% × (A))	212 452	217 205	222 380	227 112	231 820
(C) Patients who will get a repeat screen in the next year (=(A)−(B))	1 421 796	1 453 606	1 488 238	1 519 901	1 551 408
(D) Number of screens that are repeat annual screens (=(C) from earlier year)	NA	1 421 796	1 453 606	1 488 238	1 519 901
(E) Number of screens that represent new patients receiving baseline screens (=(A)−(D))	(All)	249 015	257 012	258 775	263 327
					
*Summary of the breakout of baseline vs annual repeat screens per year:*					
Patients receiving baseline screen (all in year 1;=(E) in subsequent years):	1 634 248	249 015	257 012	258 775	263 327
Patients receiving annual repeat screen (none in year 1;=(D) in subsequent years):	0	1 421 796	1 453 606	1 488 238	1 519 901
Total Screened Population (sum of baseline and annual repeat screens):	1 634 248	1 670 811	1 710 618	1 747 012	1 783 228

a The values shown above for total screened population are based on the ‘Input 1’ column in Table 1. For simplicity, values generated using the columns labelled ‘Input 2’ and ‘Input 3’ values are not shown here. Refer to Table 1 and Table 2.

b For simplicity, the values associated with the cohort ages 60–79 years are not shown here.

**Table 4:** Percentage of patients receiving each screening procedure in the baseline screen

**I-ELCAP baseline screening results** a		**Patients**	**% of total**	**EDCTS screening procedures that would be applied to the nodules sizes identified by I-ELCAP**	
(A)	Patients receiving initial CT scan:	1000	100	(A)	Receive MDCT
					
*Positive non-calcified nodules identified:*					
(B)	Nodules <=5 mm	136	13.6	(B)	MDCT in 1 year^b^
(C)	Nodules 6–10 mm, negative upon workup	56	5.6	(C)	MDCT in 3 months, assume no growth and therefore additional MDCT in 1 year^b^
(D)	Nodules 6–10 mm, positive upon workup	14	1.4	(D)	MDCT in 3 months, assume growth and therefore begin treatment^b^
(E)	Nodules 11–20 mm, negative upon workup	14	1.4	(E)	Receive PET, assume negative and therefore additional MDCT in 3 months
(F)	Nodules 11–20 mm, positive upon workup	8	0.8	(F)	Receive PET, assume positive and therefore perform FNAB
(G)	Nodules >20 mm	5	0.5	(G)	All receive FNAB

Abbreviations: EDCTS=early detection approach using computed tomography screening; FNAB=fine-needle aspiration biopsy; MDCT=multidetector computed tomography; PET=positron emission tomography.

a Reference Henschke *et al* (1999).

b For purposes of this screening protocol, FNAB and PET are not utilised for nodules ⩽10 mm in size.

**Table 5:** Percentage of patients receiving each screening procedure in the annual repeat screen

**I-ELCAP annual repeat screening results** a		**Patients**	**% of total**	**EDCTS screening procedures that would be applied to the nodules sizes identified by I-ELCAP**	
(A)	Patients receiving initial CT scan:	1184	100	(A)	Receive MDCT
					
*Positive non-calcified nodules identified:*					
(B)	Nodules ⩽5 mm, negative upon workup	7	0.6	(B)	MDCT in 3 months, assume no growth^b^
(C)	Nodules ⩽=5 mm, positive upon workup or not ruled out	9	0.8	(C)	MDCT in 3 months, assume growth and therefore patient receives treatment (no further screening procedures)^b^
(D)	Nodules 6–10 mm, negative upon workup	7	0.6	(C)	MDCT in 3 months, assume no growth and therefore additional MDCT in 1 year^b^
(D)	Nodules 6–10 mm, positive upon workup or not ruled out	9	0.8	(D)	MDCT in three months, assume growth and therefore begin treatment^b^
(E)	Nodules 11–20 mm, negative upon workup	3	0.3	(F)	Receive PET, assume negative and therefore additional MDCT in 3 months
(F)	Nodules 11–20 mm, positive upon workup or not ruled out	4	0.3	(E)	Receive PET, assume positive and therefore perform FNAB
(G)	Nodules >20 mm	3	0.3	(G)	All receive FNAB

Abbreviations: EDCTS=early detection approach using computed tomography screening; FNAB=fine-needle aspiration biopsy; MDCT=multidetector computed tomography; PET=positron emission tomography.

a Reference Henschke *et al* (2001).

b For purposes of this screening protocol, FNAB and PET are not utilised for nodules ⩽10 mm in size.

**Table 6:** Comparison of the total number of diagnosed tumours between the STID and EDCTS methods

**Cohort ages 40–79 years** a				
**Scenario code (includes all scenario codes**	**Total number of diagnosed tumours (5-year period)**		**Increase in**	**% Increase in**
**beginning with the first two letters indicated)** b	**STID**	**EDCTS** c	**EDCTS tumours**	**EDCTS tumours**
AD…		86 818	31 357	57
AE…		73 323	17 862	32
AF…		82 849	27 388	49
BD…	55 461	92 044	36 583	66
BE…	(Same for all scenarios)	76 300	20 839	38
BF…		87 414	31 953	58
CD…		81 592	26 131	47
CE…		70 346	14 885	27
CF…		78 284	22 823	41

Abbreviations: EDCTS=early detection approach using computed tomography screening; STID=symptomatic tumour identification.

a Applying the same analysis to the cohort ages 60–79 years (not shown here), the increase in tumours identified under the EDCTS method ranged from 24 to 60%.

b The scenario codes represent the combination of input variables and associated codes from Table 1.

c The estimated number of EDCTS tumour varies depending on the following input parameters: (1) Estimated percentage of patients with a 10 pack-year history of smoking; and (2) the estimated percent of patients complying with screening recommendations. As such, the EDCTS number of diagnosed tumours will vary for scenario codes representing different values for these input parameters

**Table A1:**
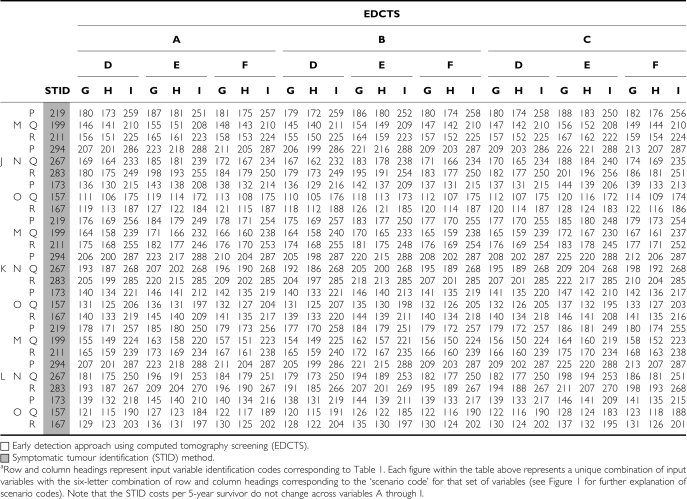
Summary of the cost per 5-year survivor for all 729 scenarios^a^ analysed for the cohort ages 40–79 years (2005 USD, in thousands)

**Table A2:**
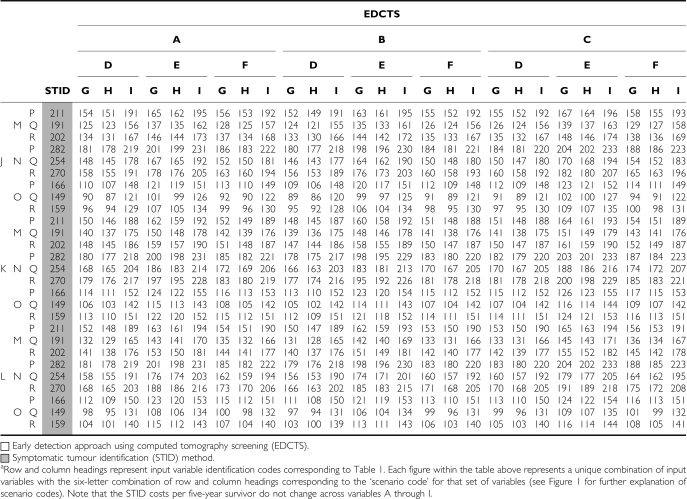
Summary of the cost per 5-year survivor for all 729 scenarios^a^ analysed for the cohort ages 60–79 years (2005 USD, in thousands)

**Table A3:**
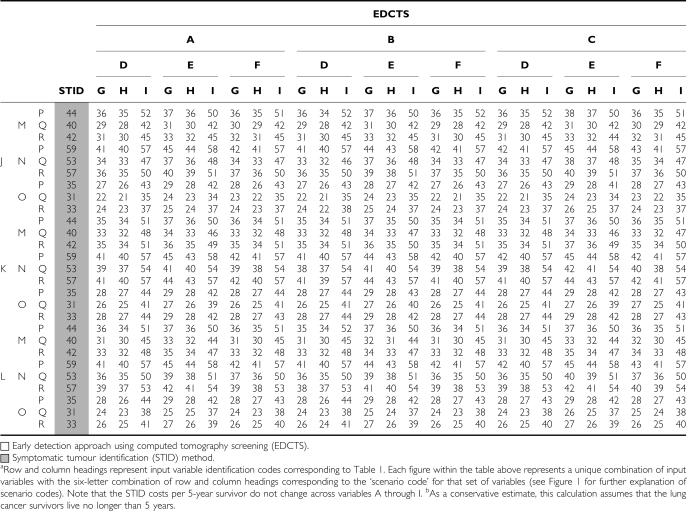
Summary of the cost per lung cancer survivor year of life for all 729 scenarios^a^ analysed for the cohort ages 40–79 years (2005 USD, in thousands)^b^

**Table A4:**
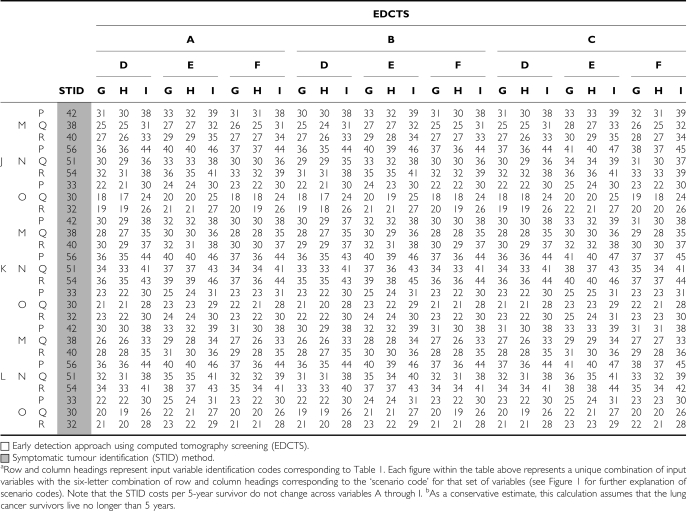
Summary of the cost per lung cancer survivor year of life for all 729 scenarios^a^ analysed for the cohort ages 60–79 years (2005 USD, in thousands)^b^

## Appendix

See Tables A1, A2, A3, A4
